# Diabetes Life Expectancy Prediction Model Inputs and Results From Patient Surveys Compared With Electronic Health Record Abstraction: Survey Study

**DOI:** 10.2196/44037

**Published:** 2023-11-09

**Authors:** Sean Bernstein, Sarah Gilson, Mengqi Zhu, Aviva G Nathan, Michael Cui, Valerie G Press, Sachin Shah, Parmida Zarei, Neda Laiteerapong, Elbert S Huang

**Affiliations:** 1 Rush University Medical Center, Chicago, IL, United States; 2 Section of General Internal Medicine, Department of Medicine, University of Chicago, Chicago, IL, United States; 3 College of Medicine, University of Illinois Chicago, Chicago, IL, United States

**Keywords:** diabetes mellitus, patient-reported outcome measure, life expectancy, diabetes, diabetic, predict, model, mortality, chart review, chart abstraction, patient chart, prediction model, patient-reported outcome

## Abstract

**Background:**

Prediction models are being increasingly used in clinical practice, with some requiring patient-reported outcomes (PROs). The optimal approach to collecting the needed inputs is unknown.

**Objective:**

Our objective was to compare mortality prediction model inputs and scores based on electronic health record (EHR) abstraction versus patient survey.

**Methods:**

Older patients aged ≥65 years with type 2 diabetes at an urban primary care practice in Chicago were recruited to participate in a care management trial. All participants completed a survey via an electronic portal that included items on the presence of comorbid conditions and functional status, which are needed to complete a mortality prediction model. We compared the individual data inputs and the overall model performance based on the data gathered from the survey compared to the chart review.

**Results:**

For individual data inputs, we found the largest differences in questions regarding functional status such as pushing/pulling, where 41.4% (31/75) of participants reported difficulties that were not captured in the chart with smaller differences for comorbid conditions. For the overall mortality score, we saw nonsignificant differences (*P*=.82) when comparing survey and chart-abstracted data. When allocating participants to life expectancy subgroups (<5 years, 5-10 years, >10 years), differences in survey and chart review data resulted in 20% having different subgroup assignments and, therefore, discordant glucose control recommendations.

**Conclusions:**

In this small exploratory study, we found that, despite differences in data inputs regarding functional status, the overall performance of a mortality prediction model was similar when using survey and chart-abstracted data. Larger studies comparing patient survey and chart data are needed to assess whether these findings are reproduceable and clinically important.

## Introduction

Prediction models are being increasingly used in many aspects of clinical practice to identify distinct patient subpopulations and to guide the selection of therapies for chronic disease management [1,2]. For instance, among older adults with diabetes, life expectancy prediction has become integral for determining individualized glycated hemoglobin (HbA_1c_) goals [3]. The benefits of intensive glucose control (eg, HbA_1c_ level <7.0%) are not realized for 9 to 10 years, and the risks, such as hypoglycemia and falls, in patients with limited life expectancy generally outweigh potential benefits [4-6]. Based on this comparison of life expectancy and time to benefit from intensive glucose control, multiple diabetes care guidelines have recommended the individualization of HbA_1c_ goals by health status [4].

Many prediction models are designed to rely on readily available data from electronic health records (EHRs) and insurance claims [1,7-9]. However, major life expectancy prediction models for older patients require self-reporting of their ability to perform basic and instrumental activities of daily living [10,11]. Some of these variables may already exist in various structured or unstructured elements of the EHR, but prior studies have demonstrated that patient-reported outcomes (PROs) obtained via survey may differ from data obtained from the patient’s EHR [12-15].

Despite increasing calls to use prediction models, the optimal approach to collecting the inputs for these models as part of clinical practice is unclear. An important question is whether we can rely solely on existing EHR data to populate these models or if we should expend additional resources to systematically collect PRO data. EHRs can be readily available for data extraction and analysis. In addition, EHRs have been designed to include fields for PRO data during the course of routine care. However, systematically collected PROs may reduce rates of missing data, have the benefit of coming directly from the patient, and can be tailored to the needs of the survey or model. Collecting the data often requires more time and effort due to tool development, management of patient follow-up times and nonresponse rates, and addressing of patient difficulties in responding due to cognitive, physical, technological, or other reasons [16]. Thus, it is important to determine the value of systematic data collection for PROs.

While conducting the My Diabetes GOAL (MDG) pilot trial, we systematically collected data for a prediction model via an electronic survey from older patients with diabetes. This created an opportunity to compare results from a life expectancy model using data from chart abstraction and data from patient surveys. The aims of this study were to (1) characterize the magnitude of variation in the availability of data from chart abstraction or survey for individual variables and (2) determine any difference in mortality risk score or life expectancy prediction for patients based on surrogate information from chart abstraction compared to survey.

## Methods

### Study Population

University of Chicago Medicine (UCM) is an urban academic medical center in Chicago, IL, serving a predominantly Black patient population. Study participants were recruited from UCM’s primary care clinic from June 2018 to December 2019 to participate in the MDG pilot, which was a delayed comparator study that creates personalized goals using patient responses and a predictive model. All participants were ≥65 years of age, had a confirmed diagnosis of type 2 diabetes mellitus in the medical record, had attended at least 1 outpatient primary care clinic visit within the year prior to recruitment, and had an active online portal account through UCM. Throughout the MDG pilot, patients were surveyed via the portal about their health status. All participants who completed the initial survey, regardless of the MDG randomization arm to which they were allocated, were included in this study.

### Ethical Considerations

The study protocol was approved by the institutional review board at UCM (number 18-0425), and all participants provided written informed consent.

### Mortality Index Score

The mortality index deployed during this pilot study (hereafter referred to as the Lee Index) was developed and validated using data from the Health and Retirement Study [17]. The index has been widely used in other geriatric studies [18-22]. The index incorporates demographic characteristics (age, sex, and BMI), information about specific self-reported disease diagnoses (presence of cancer or malignant tumor, excluding skin cancer; presence of heart failure; an activity-limiting lung condition or home oxygen use; and recent cigarette smoker), and self-reported performance on a series of functional limitations (difficulty bathing or showering, difficulty managing money, difficulty ambulating several blocks, and difficulty pushing or pulling large objects). Points assigned to various answers to each question range from 1 to 7 out of a total of 26. The Lee Index calculation was performed twice for each patient: first using the information provided by the patient in their online portal survey and then using the data obtained retrospectively from the chart review described below. The scores were categorized into the following American Diabetes Association (ADA) classes for older adults with diabetes: participants with scores 0 to 7 were classified into class 1 with an HbA_1c_ goal <7.5%, scores 8 to 11 into class 2 with an HbA_1c_ goal <8.0%, and scores >12 into class 3 with an HbA_1c_ goal <8.5% [23,24]. These score cutoffs correspond with life expectancies of >10 years, 5 to 10 years, and <5 years.

### Survey

As part of the enrollment and intervention for the larger MDG study, a comprehensive survey was sent to all study participants via an online patient portal in the EHR with questions about their individual health, diabetes management, treatment preferences, and adverse events. A subset of survey answers corresponded to the mortality index questions that were used to calculate the survey score.

### Chart Review

A research assistant performed a retrospective chart review in UCM’s EHR for all study participants over 6 months prior to and through enrollment. Relevant data were systematically obtained. The medical history and problem list sections were reviewed for relevant diagnoses. Demographic data were obtained from registration forms. Body measures were found in encounter flowsheets. Functional conditions, limitations, and any assessments or clarification of diagnoses were found by manually searching for keywords in a note search feature. All data obtained were recorded and coded into an electronic database. Differences between all survey responses and chart review were compared and frequencies were summed to determine which questions had the greatest diferences between the survey and the chart review.

### Life Expectancy Prediction

Life expectancy predictions were calculated from the Gompertz-Predicted Median Life Expectancy data based on the Lee Index score calculated both from the survey and the chart review [11].

### Data Analysis

All quantitative outcomes were summarized with descriptive statistics. The McNemar test was used to evaluate the differences in patients’ individual data inputs and ADA classes between chart abstraction and the survey. The Wilcoxon signed-rank test was used to evaluate the differences in patients’ mortality index scores and life expectancy between chart abstraction and the survey. Linear regression was also used to model the correlation between the mortality index scores from chart abstraction and the survey. RStudio (version 4.1.0; Posit PBC) was used for data analysis.

## Results

### Survey

There were 75 participants who completed the survey through the larger MDG study. The majority of participants were female (n=50, 66.7%), identified as Black (n=49, 65.3%), and had a mean age of 72.5 (SD 5.3) years. Additionally, most participants (n=59, 78.7%) had an HbA_1c_ level ≤8%, while approximately a quarter (n=17, 22.7%) had an HbA_1c_ level <6.5% prior to enrollment (Table 1).

**Table 1.** Participant characteristics from chart abstraction at 6 months prior to randomization (N=75).

Characteristic			Value
**Age (years)**			
	Mean (SD)		72.5 (5.3)
	Median (IQR)		72 (6)
**Sex, n (%)**			
	Female		50 (66.7)
	Male		25 (33.3)
**Race, n (%)**			
	Black		49 (65.3)
	White		22 (29.3)
	Other^a^		4 (5.3)
**HbA_1c_^b^^c^**			
	Mean (SD)		7.1 (1.0)
	Median (IQR)		6.9 (1.2)
	**Level, n (%)**		
		<9%	65 (86.7)
		<8%	59 (78.7)
		<6.5%	17 (22.7)
**Blood pressure (mm Hg)**			
	**Systolic**		
		Mean (SD)	131 (16.7)
		Median (IQR)	130 (21)
	**Diastolic**		
		Mean (SD)	68 (10.2)
		Median (IQR)	67 (12)
**BMI (kg/m** ^**2**^ **)**			
	Mean (SD)		32 (7.1)
	Median (IQR)		31 (8.7)
	<25 kg/m^2^, n (%)		11 (14.7)
**Use of diabetic medications, n (%)**			
	Metformin		49 (65.3)
	Insulin		22 (29.3)
	Sulfonylureas		15 (20)
	GLP-1^d^ agonists		12 (16)
	SGLT2^e^ inhibitors		5 (6.7)
**Other characteristics, n (%)**			
	Statin use		63 (84)

a Participants identified as “Asian Indian” (n=3) and “More than one race; Hispanic” (n=1).

b HbA_1c_: glycated hemoglobin.

c No recent HbA_1c_ (n=8).

d GLP-1: glucagon-like peptide 1.

e SGLT2: sodium-glucose cotransporter-2.

### Individual Survey Data Inputs

**Figure 1.**
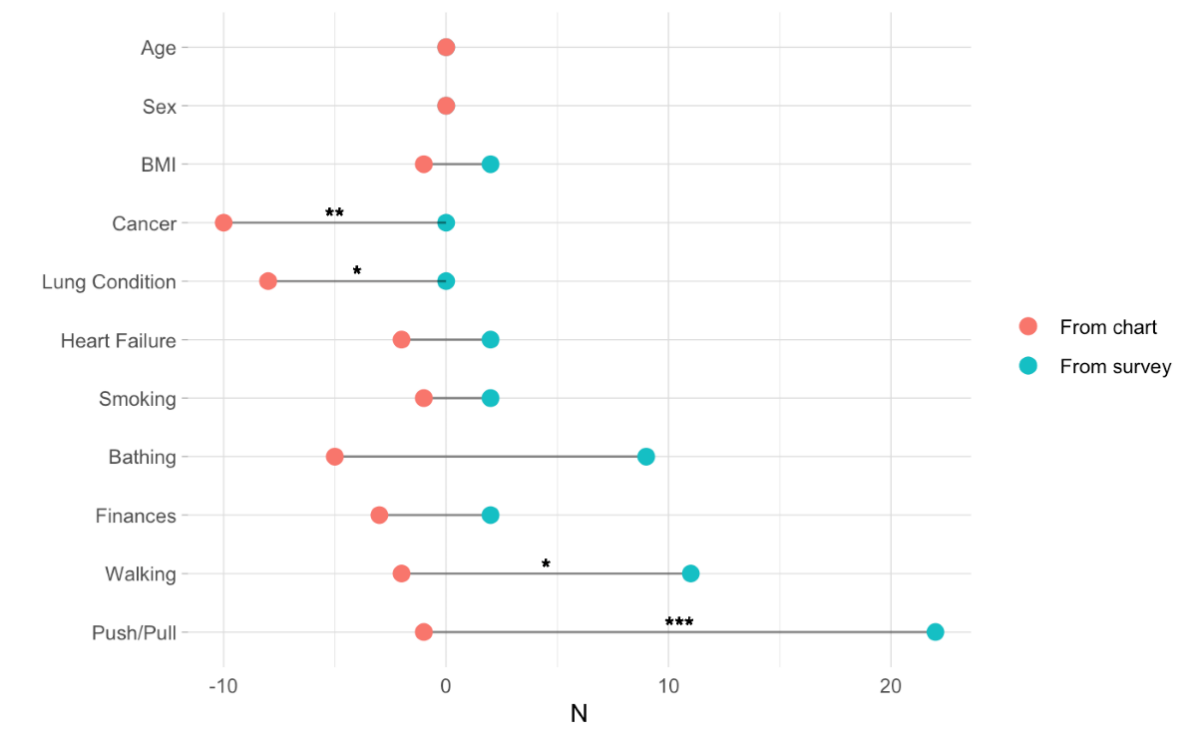
Differences in Lee Index data inputs. The x-axis reflects the number of participants across the study with concordance or discordance across survey and chart-based data inputs. Zero represents agreement between individual inputs. “N*”* refers to the number of participants with a discordant response to a question. A positive N indicates that the response to the survey question was positive compared to the chart, and a negative N indicates that the response to the chart question was positive compared to the survey. Asterisks indicate a significant difference between the chart and the survey (*P<.05, **P<.01, ***P<.001).

Individual responses to questions were compared between the 2 methods and frequencies were summed to show notable differences across all questions except for demographics (Figure 1). Across questions, the largest differences in available data were related to physical function. A total of 31 (41.3%) individuals noted difficulty completing a task (ie, “bathing or showering,” “walking a few blocks,” and “pushing or pulling large objects”) in the survey that was not captured in the chart review. A total of 23 (30.7%) participants had differences in their ability to push and pull; of these, 22 (95.7%) reported difficulties not found in the chart and only 1 had a reported difficulty in the chart that was not found in the survey. The last functional question, regarding difficulty with finances, differed for 5 participants where more disability was identified in the chart review compared to the survey.

Additionally, 10 participants had a “cancer or a malignant tumor” identified by the chart review that was not reported in the survey, and 8 participants had “a lung condition that limits [their] usual activities” identified by their chart review that was not reported in the survey.

### Mortality Index Score

**Figure 2.**
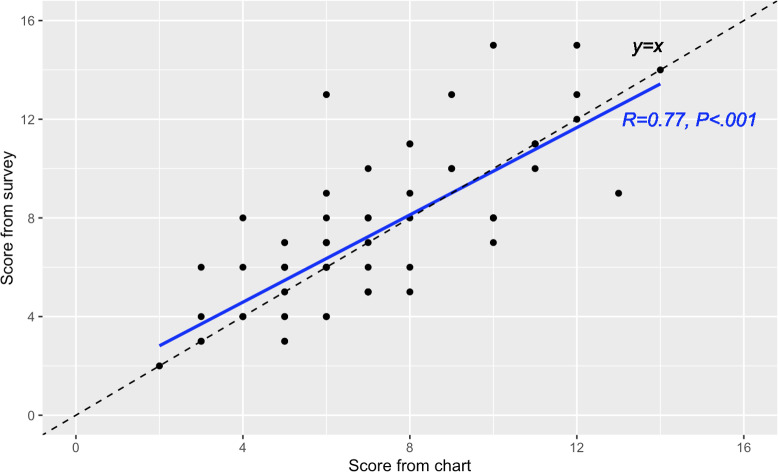
Mortality index score from the survey plotted against the abstracted score from the chart.

The average mortality index score for all patients based on information from the self-reported MDG survey was 7.23 (SD 3.04, IQR 4), while the score calculated from the chart abstraction was 7.07 (SD 2.66, IQR 4). The difference between the Lee Index score assignment comparing survey and chart review was not statistically significant (*P*=.82). Comparing individual participants’ scores, there was a significant positive correlation between the scores (*R*=0.77) (Figure 2).

### Life Expectancy Prediction

Using the mortality scores, the mean life expectancy prediction for all patients based on information from the MDG survey was 13.37 (SD 6.03, IQR 8.9) years compared to 13.40 (SD 5.53, IQR 8.9) years from the chart abstraction (*P*=.82).

### ADA Class

**Figure 3.**
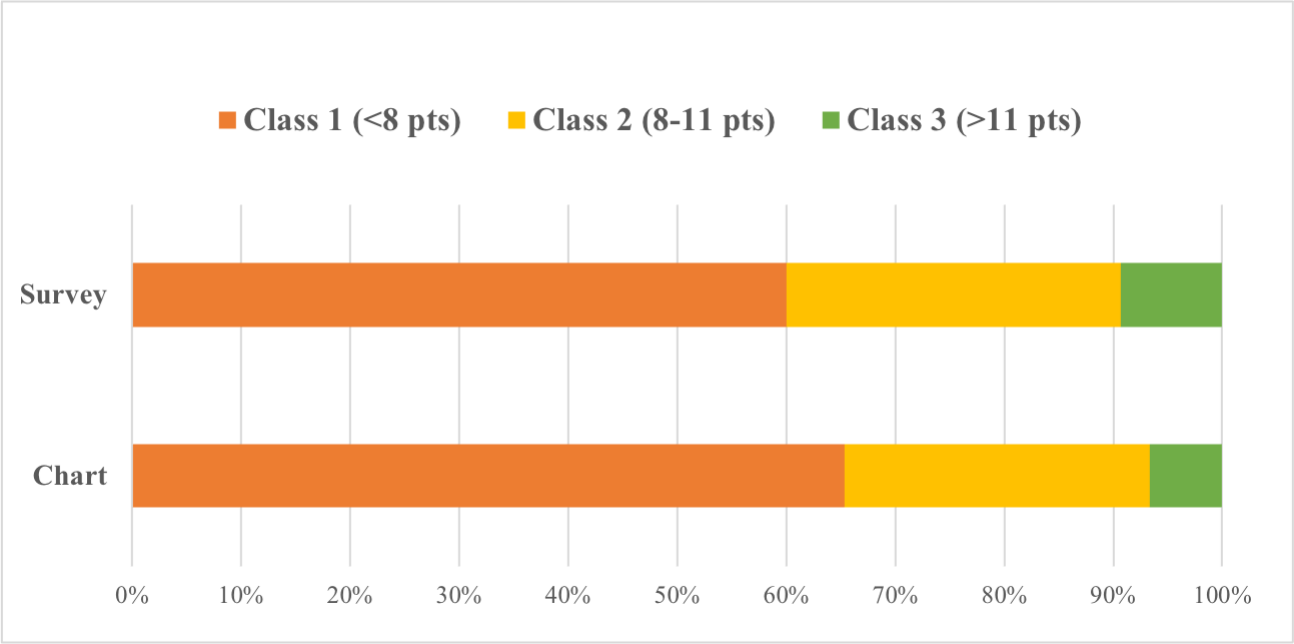
Class comparison. Class was calculated from the inputs for the Lee Index score as a distribution of the total population. Pts: points.

Of the 75 participants, 15 (20%) were placed in different ADA classes based on comparison of the survey and the chart review (*P*=.54; Table 2). The class distribution of the population was similar and not significantly different between the 2 class calculations (*P*=.74; Figure 3). From the survey, 60% (n=45) were in class 1, 31% (n=23) were in class 2, and 9% (n=7) were in class 3. The chart review percentages were 65% (n=49), 28% (n=21), and 7% (n=5), respectively.

**Table 2.** Class assignment based on the scores of the Lee Index from survey and chart review data.

Class assignment	Survey class 1, n	Survey class 2, n	Survey class 3, n
Chart class 1	41	7	1
Chart class 2	4	15	2
Chart class 3	0	1	4

## Discussion

### Principal Findings

In this small exploratory study, we found differences in the reported functional status and disease history of patients when comparing data from chart reviews and surveys. Despite these differences in data inputs, the overall model life expectancy prediction was not significantly different across the chart review and the patient survey. It is not clear from this exploratory study whether the differences in data availability from the chart and the patient survey would actually lead to clinically significant differences in glucose control recommendations for older patients at scale. An alternative interpretation is that available data in the EHR performed reasonably well despite data limitations.

With the exception of demographics, other domains had differences between the chart review and the survey, the largest difference being in the functional status domains. The functional question related to having trouble pushing or pulling showed the largest difference between the 2 abstraction methods. The same question had the least predictive power in the calculations so it did not affect the overall score and subsequent class calculations significantly in this model. In models for other clinical conditions such as frailty, questions regarding functional status could have greater impact on the accuracy of predictions [25].

An additional area of difference was related to diagnoses. We found that many participants underreported temporally distant and low-stage cancers or early-stage chronic medical conditions such as heart failure or lung disease compared to what was documented in the chart.

A notable limitation in this study is the study size, which likely leaves the study underpowered to identify significant differences. Another is that our original study required patients to be able to use our patient portal application, MyChart. Thus, patients were excluded from recruitment if they did not have an active MyChart account. It is possible that patients who have an active patient portal are more engaged in care and have a more complete EHR than patients without an active portal.

We continued to see differences in several health domains as has been noted previously [15], with the largest differences observed in questions related to functional assessments. Since functional status is clinically important for an array of decisions, our study suggests the need to systematically capture functional status data in the EHR. As a health industry, we already collect responses to many social and behavior questions, like smoking status, to use in various models such as the ASCVD (Atherosclerotic Cardiovascular Disease) Risk Calculator [26], and effort should be made to collect more of these PROs. Our study shows that prediction models, such as the Lee Index, are incomplete without them. The overall impact of this incompleteness would be much more important for populations with significant functional impairments.

### Conclusions

The future of health care will likely continue toward an increasing use of prediction models. As health care systems incorporate these models, there will be questions about whether or not to incorporate models that include PROs as inputs. Our study suggests that some of these models may actually already perform reasonably well with available EHR data despite some degree of missingness. Whether or not the degree of missing data leads to clinically important differences will require further study of the value of systematic PRO data collection. Tools such as MyChart open the door for routine collection of such patient-reported data at a reasonable cost to health systems. As systems become more experienced with systematically collecting PROs, these data become part of the EHR. Beyond the utility for predicting future outcomes, eliciting PROs may also have a benefit for patient engagement and health behavior change.

## Abbreviations

ADA: American Diabetes Association
ASCVD: Atherosclerotic Cardiovascular Disease
EHR: electronic health record
HbA_1c_: glycated hemoglobin
MDG: My Diabetes GOAL
PRO: patient-reported outcome
UCM: University of Chicago Medicine
